# Bayesian hypothesis testing for human threat conditioning research: an introduction and the condir R package

**DOI:** 10.1080/20008198.2017.1314782

**Published:** 2017-05-16

**Authors:** Angelos-Miltiadis Krypotos, Irene Klugkist, Iris M. Engelhard

**Affiliations:** ^a^ Department of Clinical Psychology, Faculty of Social and Behavioural Sciences, Utrecht University, Utrecht, The Netherlands; ^b^ Department of Methodology and Statistics, Faculty of Social and Behavioural Sciences, Utrecht University, Utrecht, The Netherlands; ^c^ Department of Research Methodology, Measurement and Data Analysis, Faculty of Behavioural, Management and Social Sciences, Twente University, Enschede, The Netherlands

**Keywords:** Post-Traumatic Stress Disorder, Bayes factor, experimental psychopathology, fear, treatment

## Abstract

Threat conditioning procedures have allowed the experimental investigation of the pathogenesis of Post-Traumatic Stress Disorder. The ﬁndings of these procedures have also provided stable foundations for the development of relevant intervention programs (e.g. exposure therapy). Statistical inference of threat conditioning procedures is commonly based on *p*-values and Null Hypothesis Signiﬁcance Testing (NHST). Nowadays, however, there is a growing concern about this statistical approach, as many scientists point to the various limitations of *p*-values and NHST. As an alternative, the use of Bayes factors and Bayesian hypothesis testing has been suggested. In this article, we apply this statistical approach to threat conditioning data. In order to enable the easy computation of Bayes factors for threat conditioning data we present a new R package named condir, which can be used either via the R console or via a Shiny application. This article provides both a non-technical introduction to Bayesian analysis for researchers using the threat conditioning paradigm, and the necessary tools for computing Bayes factors easily.

Threat conditioning^1^ is one of the dominant paradigms in the experimental study of Post-Traumatic Stress Disorder (PTSD) (Beckers, Krypotos, Boddez, Effting, & Kindt, 2013; for relevant examples see Orr et al., 2000; Sijbrandij, Engelhard, Lommen, Leer, & Baas, 2013). This procedure typically involves the pairing of an initially neutral stimulus (e.g. picture of a square; Conditioned Stimulus or CS) with an evolutionary aversive stimulus or event (e.g. shock administration; Unconditioned Stimulus or US). As a result of this procedure, the CS will come to evoke threat/fear responses (e.g. higher level of skin conductance; Conditioned Responses or CRs) even in the absence of the US. Threat conditioning has proven particularly useful in uncovering the working mechanisms behind the acquisition of PTSD symptomatology, with a number of theories suggesting that associations between primarily innocuous stimuli (e.g. visit to a shopping centre) with aversive stimuli or events (e.g. a terrorist attack) lie at the core of the pathogenesis of, among others, trauma-related disorders (Bouton, Mineka, & Barlow, 2001; Mineka & Zinbarg, 2006). Conditioning procedures have provided the basis for the development of therapeutic interventions for the reduction of PTSD symptomatology. To illustrate, the roots of exposure therapy, one of the commonly used interventions for PTSD, are found in the conditioning procedure of threat extinction (Eddy, Dutra, Bradley, & Westen, 2004; Foa, Steketee, & Rothbaum, 1989).

As in most experimental and applied branches in psychology, statistical inference of threat conditioning procedures is based on *p*-values and Null Hypothesis Significance Testing (NHST). Despite its popularity, this statistical approach has long been heavily criticized, with many pointing to its serious shortcomings (e.g. Cohen, 1994; Wagenmakers, 2007).

Here we present an alternative type of statistical approach that overcomes most, yet not all, shortcomings of *p*-values and NHST. This approach is based on the computation of Bayes factors. To further encourage the use of Bayesian analysis in threat conditioning research, we also present our recently developed R package named condir. Our package allows the easy analysis of (threat) conditioning data, with a minimal amount of effort. Collectively, this article provides a non-technical introduction to Bayesian analysis, especially for researchers working with the threat conditioning paradigm, and a tool for computing the relevant Bayes factors easily.

## 1. p-values and NHST for threat conditioning data

Let us consider a common differential threat conditioning experiment (see Orr et al. 2000 for an example with PTSD and non-PTSD individual). Such experiments typically begin with the baseline assessment of CRs (e.g. fear potential startle) by presenting singly two CSs: the CS1 and the CS2. Because no pairing with the US has taken place, no differences are expected in CRs during CS presentation. The threat acquisition phase follows, during which the CS1 is paired with US-presentation, whereas the CS2 is paired with US-omission. At the end of threat acquisition, stronger CRs to the CS1, compared to the CS2, are expected. Usually, experiments also include a threat extinction procedure, during which the CSs are not followed by a US, at the end of which similar CRs towards the CSs typically emerge.

Within a NHST framework, the analyses plan would entail carrying out three paired sample *t*-tests, one for each phase, where CRs between the CSs are compared. Then, and separately for each phase, a *p*-value below a predefined 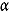 level (e.g. .05, or .016 if a Bonferroni correction for multiple testing is used) would be taken as evidence for presence of differences in CRs towards the two CSs, whereas a *p*-value above or equal to that level as failure to acquire evidence for differences in CRs towards the two CSs.

As mentioned above, however, this statistical approach conveys serious shortcomings (see Cohen, 1994; Krueger, 2001; Krypotos, Blanken, Arnaudova, Matzke, & Beckers, in press; Loftus, 1996; Trafimow, 2003; Wasserstein & Lazar, 2016; Wagenmakers, 2007 for extensive discussions). To name a few within a NHST, it is hard to quantify evidence for the null hypothesis, the stopping plan influences the direction of the results (i.e. whether *p*-values cross the 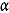 level), and inferences are based on unobserved data (Cohen, 1994; Wagenmakers, 2007). Given those limitations, many have suggested alternative approaches such as model selection (Tibshirani, 1996; Yuan & Lin, 2006) or Bayesian statistics (Dienes, 2016; Wagenmakers, Morey, & Lee, 2016; Wagenmakers et al., 2017). Although each approach comes with pros and cons, here we extend on Bayesian hypothesis testing, a Bayesian alternative to NHST, and how it could be particularly useful in analysing conditioning data.

## 2. Bayesian hypothesis testing for threat conditioning data

To better explain Bayesian hypothesis testing, let us consider the study by Krypotos, Arnaudova, Effting, Kindt, and Beckers (2015) that used an action tendencies bias modification procedure for reducing conditioned threat responses. On the first day, participants underwent a standard threat acquisition procedure, with one stimulus (i.e. CS1) always being followed by electric stimulation and the other stimulus (i.e. CS2) never being followed by electric stimulation. On the following day, participants underwent a threat extinction procedure, an action tendencies modification procedure, and a reinstatement procedure.

For each CS separately, Krypotos et al. (2015) collected US-expectancy, skin conductance ratings, startle reflex, and valence ratings. The full data set is available at https://osf.io/3apxv/, and the original analyses of these data are reported in Krypotos et al. (2015). Since here we use this data set for illustrative purposes, we analyse only a portion of the data. Specifically, we use the startle reflex data collected during the threat acquisition phase. The startle reflex data were collected separately for each trial (i.e. eight CS1 and eight CS2 trials). In line with Krypotos et al. (2015), we analysed average startle reflex data across acquisition trial and separately for each CS. To replicate the results we report here, follow the analyses steps in Section 3.

### 2.1 D*efinition of Bayesian hypotheses*

In the case of hypothesis testing, Bayesian analysis proceeds as in Equation (1):

(1)
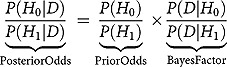

First, one needs to define two competing hypotheses (e.g. the 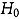 and the 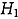) prior to taking any collected data into account. For our example, the 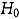 would be defined as the absence of an effect (e.g. no differences between CRs during the presentation of the CSs or 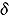 = 0; where 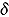 is the effect size of the population), whereas the 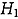 would be defined as the presence of an effect (e.g. differences between CRs during the presentation of the CSs or 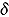 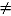 0). The relative plausibility of these hypotheses prior to seeing the data is the so-called *prior odds*. For example, at the end of a threat acquisition phase, one could expect that the odds should be higher for the 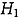, indicating differences in CRs, than for the 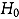. However, in Bayesian hypothesis testing, those odds are often set to 1, indicating equal odds for both hypotheses (Dienes, 2016; Rouder, Speckman, Sun, Morey, & Iverson, 2009). In our example, those odds were also set to 1.

### 2.2 P*rior distributions*

The second step in Bayesian hypothesis testing is the definition of a *prior probability distribution* for each model parameter (here the 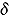 parameter). These prior distributions quantify our state of knowledge, before taking into account any information coming from the collected data. Prior distributions could be *uninformative*, showing no or little prior knowledge, or *informative*, which reveal prior knowledge. The selection of suitable prior distributions should be done carefully. To date, there have been efforts in developing default Bayesian tests for common statistical analyses. Let us take for example the case of the Bayesian default *t*-test (Rouder et al., 2009). In this, a Cauchy distribution is used for the 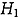 that corresponds to the values of the predicted effect sizes under this hypothesis. The form of the Cauchy distribution changes according to changes in the scale parameter, with higher numbers resulting in wider distributions. As a result, larger effects receive higher probabilities of being observed, compared to narrower distributions. For these default Bayesian tests, a scale factor of .707 or 1 is often used (see Rouder et al., 2009; Rouder & Morey, 2012; for theoretical justification of this choice of prior distribution). However, it is up to the experimenter to choose which prior distribution would be more appropriate to choose given the data at hand. For the case of threat conditioning, one could opt for different distributions based on what type of CRs were collected (e.g. psychophysiological or subjective). For example, in the meta-analysis of Duits et al. (2015), lower effect sizes were reported in the acquisition phase for psychophysiological differential responses (i.e. *d* = −.08) rather than for differential subjective responses (i.e. *d* = −.45). This piece of information can be taken into account when determining the prior distribution for the 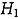 (see also van de Schoot et al., 2014, on how to determine prior knowledge in general). For our example, we used a Cauchy distribution for the 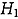 with a scale parameter to be equal to .707. This is the default value in many software programs using the Rouder et al. (2009) approach for *t*-tests. Since in Duits et al. (2015) small effect sizes were reported (i.e. *d* = −.08), we also investigated the robustness of our results by also rerunning our analyses using different scale factors (see *sensitivity analysis* section below).

### 2.3 Consideration of collected data and Bayes factors

After defining the prior distributions and the prior odds, the collected data can be considered. This leads to the updating of the relative plausibility of the competing hypotheses (i.e. *the posterior odds*). The change from the prior odds to the posterior odds is quantified by the so-called *Bayes factor*, defined as the relative likelihood of the data under the competing hypotheses. For example, a BF_10_ of 5, where the 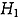 is compared to the 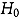, means that the data are five times more likely under the 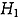 than the 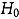. The reverse would hold for a BF_10_ of 1/5, meaning that the data are five times more likely under the 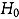 than the 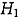. For our example, we found a BF_10_ of 6.57, meaning that it is 6.57 times more likely that there are differences between the CRs (i.e. the 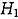) than there are not (i.e. the 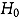). These outcomes provide strong evidence that indeed there are differences in CRs, with mean conditioned startle responses being substantially higher when the CS1 is presented than when the CS2 is presented. These Bayes factors are considered as a Bayesian alternative to NHST (Kass & Raftery, 1995; Lewis & Raftery, 1997).^2^

### 2.4 Advantages of Bayes factors over p-values and NHST

Bayes factors have a series of advantages compared to *p*-values and NHST. First, Bayes factors can quantify the relative evidence of the 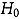 compared to the 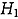 (Dienes, 2014; Rouder et al., 2009). As such, one can test whether, for example, two CSs evoke similar CRs during the extinction phase.

Second, Bayesian inference allows one to incorporate knowledge not present in the data at hand. The incorporation of such prior knowledge in statistical analyses is particularly important for threat conditioning research. This field is one of the most strongly theory-driven branches in clinical psychology. As such, threat conditioning research is most suitable for incorporating theoretical claims into the statistical analyses. Also, informative priors have proven especially useful in the field of psychotraumatology where typically small samples are collected. By having information coming from the prior distributions in addition to the collected data, Bayesian statistics can result in less biased parameter estimates compared to maximum likelihood estimation (van de Schoot, Broere, Perryck, Zondervan-Zwijnenburg, & Van Loey, 2015).

Third, Bayes factors allow one to accumulate data until enough evidence has been acquired for one of the competing hypotheses, compared to the other one (Cornfield, 1966; Deng, Lu, & Chen, 2016; Schönbrodt, Wagenmakers, Zehetleitner, & Perugini, in press). Indeed, in Bayesian analysis, more evidence usually increases support for one of the competing hypotheses, and not necessarily to a change in the direction of the results (see Schönbrodt et al., in press).

### 2.5 Challenges when using Bayes factors

Despite these advantages, there are a series of challenges that should be taken into account whenever one uses Bayes factors. First, no matter what the size of a Bayes factor may be, it cannot provide evidence for each competing hypothesis, but provides only *relative* evidence between two hypotheses (Kruschke, 2011). As noted by Dienes (2014), ‘This is already much more than *p*-values could give us’.

Second, one should be particularly careful when choosing the prior distributions of the model parameters, as different priors will by definition result in different Bayes factors. An advisable practice for detecting the influence of the choice of prior distributions to the results would be to conduct a *sensitivity analysis* (Kass & Raftery, 1995; but see Vanpaemel, 2010). In sensitivity analysis, changes in Bayes factors are reported for different choices of prior distributions. In the case of the default tests we mentioned above, one could change the scale factor of the Cauchy distribution (e.g. set it to 1.4) and compare the resulting Bayes factors after different priors have been used. Although the strength of the evidence will vary with different priors, one would hope that the direction of the Bayes factor stays the same. However, fluctuations in Bayes factors could be severe, with such fluctuations even leading to different conclusions.

Lastly, one could point to the practical difficulties when computing Bayes factors for threat conditioning data. Until recently, Bayes factors were hard to compute, requiring expertise in both mathematics and programming. To date, Bayes factors can be easily computed via web pages (e.g. www.lifesci.sussex.ac.uk/home/Zoltan_Dienes/inference/Bayes.htm, http://pcl.missouri.edu/bayesfactor), on a mouse-click basis (e.g. JASP; Love et al., 2015), or using software packages (e.g. BayesFactor, Morey & Rouder, 2015, for R). These resources have helped tremendously in the use of Bayesian analysis across scientific principles. However, they are still general-purpose programs, often requiring deep knowledge of how to correctly use them and interpret their statistical output. Although deep knowledge of the relevant algorithms is strongly advisable, sometimes researchers lack such knowledge, something that limits their analytic choices. We attempt to overcome these challenges by our software package named condir.

## 3. The condir R package

In the remainder, we show how the easier and more efficient analysis of (threat) conditioning data can be realized via condir, a new R package tailored for the statistical analyses of (threat/fear) conditioning data. Specifically, as we show below, after providing the relevant conditioning data, condir can return the corresponding results, plots, and preliminary results text in just a few lines of computer code or on a mouse-click basis. Although our package was primarily designed to enable the easy computation of Bayes factors, it can also return relevant statistics for NHST, since this is what is currently used in the threat conditioning field.

### 3.1 Installing R

The package condir requires a stable installation of R (version 3.3.2 or higher). R is a platform independent programming language and environment, freely available via the website of the Comprehensive R Archive Network (CRAN; https://cran.r-project.org/). Instructions on how to install R can be found on CRAN’s website (R Core Team, 2016).

### 3.2 Installing condir

condir is an open source program, distributed freely under the GPL-3 license. The relevant code can be found in the following GitHub repository: https://github.com/AngelosPsy/condir.

For installing condir, a R session has to be started. This is done simply by double clicking on the R icon, similar to how most software programs start across platforms. The R console will appear. Type in the following commands in the R console:

install.packages (‘condir’). Assuming a reliable internet connection, condir is now installed.

### 3.3 Using condir

After installation, the basic functions of condir can be used using the R console. In order to make our package more user friendly, we have also built a Shiny application (Chang, Cheng, Allaire, Xie, & McPherson, 2015). We now turn to how the Shiny application can be used. For the readers who are interested in the use of the R functions, within the R console, we point to the condir GitHub page (https://github.com/AngelosPsy/condir), as well as the help files within the R package.

### 3.4 Presentation of the condir Shiny application

For initiating a new analysis section, type the condir::csShine() command into the R console. After that, a window on the default internet browser of each user will appear. Alternatively, use our Shiny application by visiting https://utrecht-university.shinyapps.io/Condir/.

**Figure 1.**
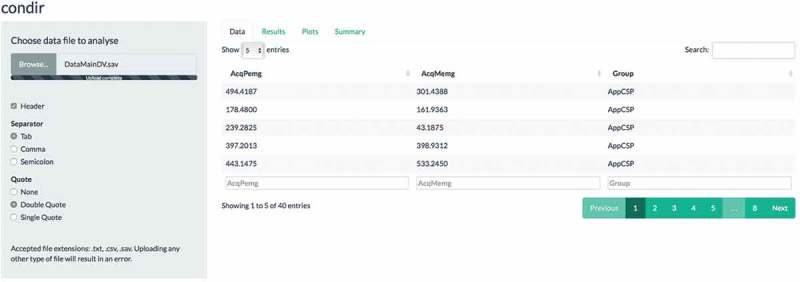
Example screen shot of the data tab of the condir Shiny app. The data refer to the main analyses we report in the main text.

To load the relevant data, click on the top left button. The user can now select the relevant data file. Currently, condir supports data files with .txt, .csv, and .sav extensions.^3^ After selecting the relevant data file and clicking ‘OK’, the data will be loaded and presented in the ‘Data’ tab (see Figure 1). For illustration, here we analyse a portion of the data by Krypotos et al. (2015) that we have described above.

**Figure 2.**
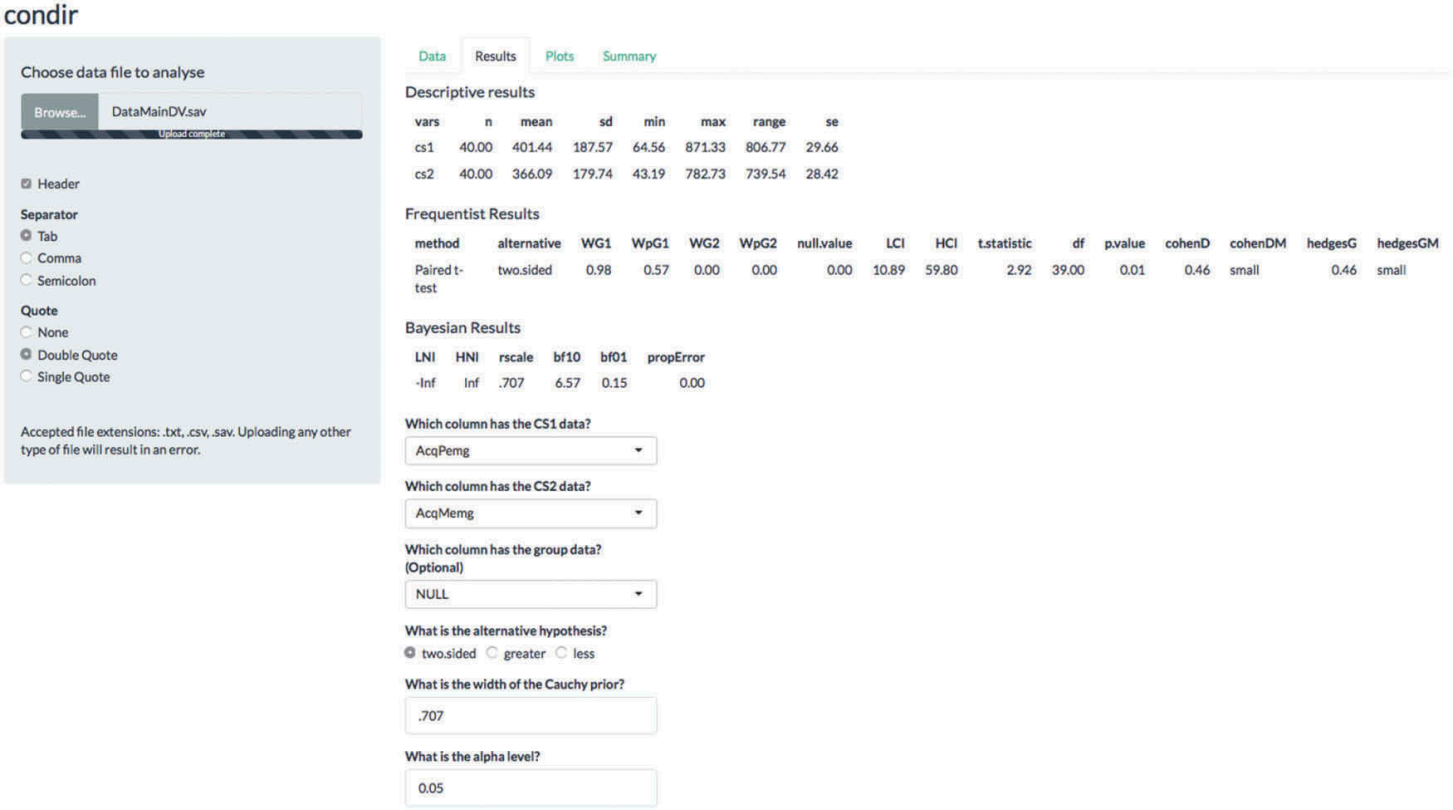
Example screen shot of the results tab of the condir Shiny app. The results refer to the main analyses we report in the main text.

By clicking on the ‘Results’ tab, the user can select which stimulus will serve as CS1 and which stimulus as CS2 (here ‘AcqPemg’ and ‘AcqMemg’ respectively; see Figure 2 for the corresponding results). By doing this, the relevant descriptive statistics are presented, together with the frequentist and Bayesian results. The frequentist results include, among others, the name of the performed test (in this case a paired samples *t*-test), the relevant *t*-statistic (i.e. 2.92), the *p*-value (i.e. .006),^4^ and the effect size (Cohen’s *d* = 0.46, small effect). Under the ‘Bayesian results’, the Bayesian results are reported for the default Bayesian paired samples *t*-test [, Speckman, Sun, MoreyIversonRouder.]. The Bayesian results include the report of the used scale factor (i.e. .707), the 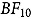 (i.e. 6.57) and the 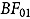 (i.e. 0.15).^5^ Of importance, the *t*-test was performed without being explicitly specified by the user; the choice was made by condir based on the provided data.

Apart from analyses between two stimuli, condir can also accommodate group analyses for up to two groups. This can be done by simply choosing which column includes the group allocation by clicking on the relevant option in the ‘Results’ tab. In the presence of groups, condir performs an independent samples *t*-test, comparing group performance to the difference in CRs for the two CSs.

**Figure 3.**
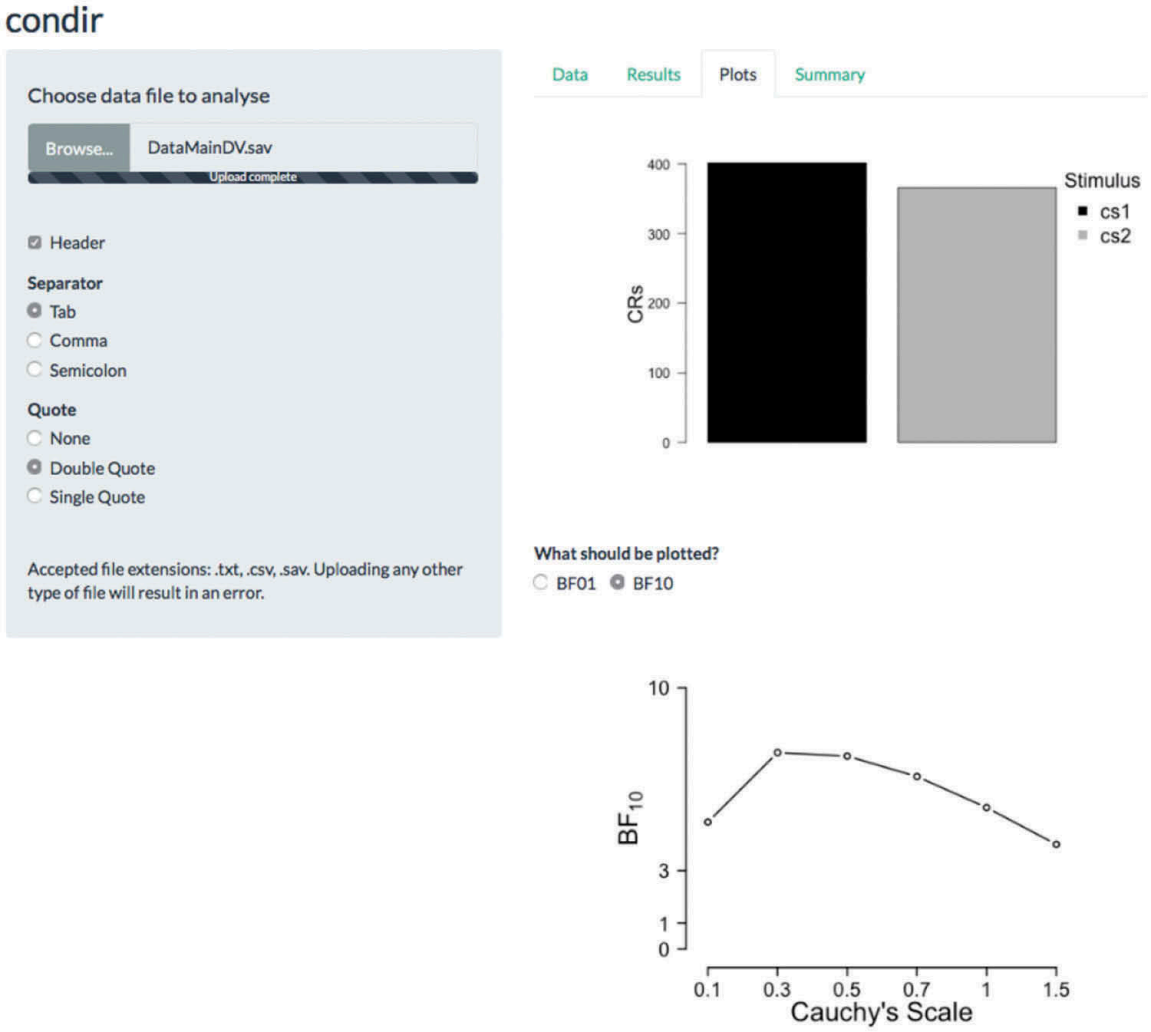
Example screen shot of the plots tab of the condir Shiny app. The ﬁgures refer to the main analyses we report in the main text.

A visualization of the variable means^6^ can be found on the top part of the ‘Plots’ panel. In Figure 3 we give an example of the plots that were generated from our main analysis. Also, for visualization of the sensitivity analysis, a robustness plot can be found on the bottom panel of ‘Plots’.^7^ There, we observe that with higher values for the scale parameter for the Cauchy distribution (see x-axis), the 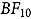 decreases after the scale factor receives values above .3 (see y-axis).^8^ For our results, we see that the higher the scaling factor of the Cauchy distribution, the less evidence for the data coming from the 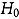 instead of the 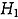. This is logical given that wider priors (in this case Cauchy priors with higher scale factors) result in more support for the 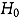 (Rouder et al., 2009).

**Figure 4.**
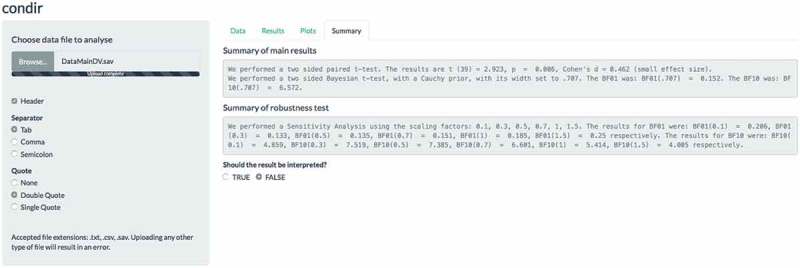
Example screen shot of the Summary tab of the condir Shiny app. The summary results refer to the main analyses we report in the main text.

Lastly, an automatic report of the results is generalized in the ‘Summary’ tab (see Figure 4 for the results of our main analysis). Specifically, in the first panel the summary of the main results (i.e. frequentist and Bayesian statistics) are presented. In the second panel, the results of the robustness test are presented. If the user desires an interpretation of the results, they can click the ‘TRUE’ option in the ‘Should the results be interpreted?’ question. This will extend the results report (not shown here) so that the *p*-values are now interpreted as significant or non-significant, and the Bayesian results are interpreted based on the categories that are suggested in the literature (for more information on Bayes factor categories, see Jeffreys, 1961, and Wetzels et al., 2011). We note that this is a report based on the numerical values. Researchers are free to question or ignore the interpretation of the outcome.

For our example the results converge, as both *p*-values and Bayes factors point towards the presence of differences between the CSs. However, this is not the case when other variables are used. For example, rerunning the analyses for the ‘ReinPemg’, as CS1, and ‘ReinMemg’, as CS2, would result in a significant *p*-value (*t*(39) = 2.185, *p* = .035, Cohen’s *d* = 0.35). Yet, the corresponding 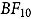 gives only small support to the data coming from the 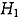 relative to the 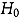 (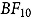 = 1.43). So, although the *p*-value suggests that there are CR differences between the two CSs, the corresponding Bayes factor provides only little support for the 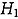, compared to the 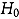. Some scientists have suggested that BFs between 1 and 3 are categorized as ‘not worth more than a bare mention’ (Jeffreys, 1961). As such, one should continue collecting data so that more evidence is accumulated for the two hypotheses, whereas the NHST results suggested that there is enough evidence to reject the 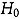.

## 4. Discussion

To date, threat conditioning researchers base their statistical inferences on *p*-values and NHST. In the first section of this paper we listed the main shortcoming of this statistical approach when analysing threat conditioning data. Then, we presented how those shortcomings can be surpassed by the adoption of BHT and Bayes factors. Specifically, and in contrast to *p*-values and NHST, the presented Bayesian approach is able to provide evidence for the 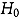 (e.g. when testing CRs at the end of a threat extinction phase), and allows the accumulation of evidence until adequate evidence has been gathered for either the 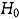 (e.g. there are no between stimuli differences) or the 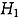 (e.g. there are differences between stimuli). As such, the adoption of Bayes factors can result in richer data conclusions; even in the case of a null result, outcomes can be interpreted, enabling the better investigation of the working mechanisms of psychopathogenesis and psychopathology reduction.

To encourage (threat/fear) conditioning researchers to use Bayesian statistics in their research, we have presented the recently developed R package, condir. As we have shown, condir can provide output for both BHT and NHST, generate the relevant plots, as well as produce a basic text of the results, with a few mouse clicks. Of importance, the tests do not need to be explicitly defined by the user, but they are automatically selected by condir. By automating many routine tasks, condir could prove to be an important tool for the more efficient analyses of threat conditioning data.

At this stage, condir can perform basic statistical analyses (i.e. paired samples *t*-test and independent samples *t*-test). More statistical tests could be added in future versions. For example, by implementing analysis of variance models in condir, conditioned responses to more than two stimuli, or across more than two groups, could be performed. condir does not provide any information about individual differences in conditioning; individual differences can be explored with more complicated statistical models than the ones presented here (see Gazendam et al., 2015). In order to encourage the further development of condir, the software code is open, allowing researchers to review and contribute with additional code. Also, any researcher can request additional features by using the issues page in GitHub (https://github.com/AngelosPsy/condir/issues). Our ultimate goal is to make condir a hub where common (Bayesian) statistical analyses for threat/fear conditioning will be stored and shared across our field.

We designed condir for the analysis of conditioning data, with minimal amount of effort. There are other statistical programs though that can prove also useful when conducting Bayesian analysis. JASP, for example, provides an excellent resource for Bayesian and frequentist statistics. At the same time, in its current form, JASP requires from the users to choose their statistical analyses and it does not generate an automatic report of the results. Other software programs (e.g. Stan or JAGS) are also powerful for Bayesian statistics but users must define the models themselves, something that requires both statistical and programming knowledge. The amount of effort needed to perform analyses for conditioning data in condir, compared to other packages, is much less, with condir being able to give a full results report (both for frequentists and Bayesian statistics), with the corresponding plots, in seconds after data input; this is much faster than the aforementioned software programs.

It should be noted that Bayesian hypothesis testing is not the only alternative for overcoming the shortcomings of NHST. For example, it has been suggested that instead of basing statistical inferences on *p*-values, one should consider effect sizes or confidence intervals (e.g. Cumming, 2014; for Bayesian alternatives to these, see Kruschke and Liddell, in press). Although useful, here we decided to focus on BHT as this is a Bayesian alternative to NHST, which is commonly used in our field. For thorough discussions of the advantages and disadvantages of different alternatives to NHST, we refer to Anderson, Burnham, and Thompson (2000); Dienes (2008); Gardner and Altman (1986) and Wagenmakers et al. (2016).

We believe that there is enough reason for threat conditioning researchers to use Bayesian analyses, instead of the commonly used statistical inference. We have provided both some key theoretical reasons for doing that, as well as a new tool that enables such analyses with a small amount of effort. For all the reasons mentioned above, we hope our research field switches to this type of inference.

## Notes

1. Note that in the literature threat conditioning is also referred to as fear conditioning.

2. Since an extended discussion of Bayesian inference is beyond the scope of this article, we point interested readers to the work by Dienes (2011), Hoijtink (2013), and Rouder et al. (2009).

3. When loading data files with .sav extension, R returns an encoding warning. This warning can be safely ignored

4. For illustrative purposes, we used a two-tailed test here, which is the default option at condir. One could also choose to use one-tailed test by clicking on the corresponding option.

5. For the complete description of what every column stands for, we refer to the help files of the ‘condir::csCompare’ in R. You can access the help page by typing in ‘?condir::csCompare’ in an R console.

6. This refers to the two variables included in the paired samples *t*-tests. In the case of the independent samples *t*-test, condir returns the means of the two variables, as well as the difference for these variables, separately for each group.

7. For the robustness plot and the sensitivity analysis, we used the default options of the BayesFactor package for determining the scale parameter of the Cauchy. Other scale parameters can be used by using the relevant functions when using condir via the R console. Note that the robustness plot is based on the equivalent plot returned when using JASP.

8. If users want to plot the , instead of the , the  can be clicked.
